# Supplementary material for: Sparse Proteomics Analysis – a compressed sensing-based approach for feature selection and classification of high-dimensional proteomics mass spectrometry data
Source: BMC Bioinformatics. 2017 Mar 9;18:160. doi: 10.1186/s12859-017-1565-4 (PMC5343371; doi:10.1186/s12859-017-1565-4)
Supplement: Additional file 1 — Supporting Information. (DOC 100 kb) [file 12859_2017_1565_MOESM1_ESM.doc]

Supporting Information	
S1 - Mass Spectrometry  Data Generation	
Chemicals, Standards and Consumables	
Gradient grade acetonitrile, ethanol, and HPLC-water were obtained from J.T. Baker	
(Phillipsburg, NJ, USA); p.a. trifluoroacetic acid (TFA) and acetone  were purchased	
from Sigma-Aldrich (Taufkirchen, Germany).  The peptide- and   protein MALDI-TOF	
calibration standards I and á-cyano-4-hydroxycinnamic acid (HCCA)   were purchased	
from Bruker Daltonics (Bremen, Germany). Automated magnetic bead preparations	
were performed using 96 well plates, TubePlates from Biozym   (Hessisch Oldendorf,	
Germany), polypropylene tubes (low profile) from Abgene (Surrey, UK),    and modular	
reservoir quarter modules from Beckman (Fullerton, USA). For  sample storage   450 µL	
CryoTubesTM were purchased from Sarstedt (Nmbrecht, Germany). Multifly needle	
sets and polypropylene serum monovettes with clotting activators were    also obtained	
from Sarstedt.	
Peptidome Separation	
All serum samples of the discovery set were processed at one time and analyzed	
simultaneously to avoid procedure-dependent errors. The external validation set was	
prepared, processed and analyzed separately. Peptidome separation of the samples was	
performed using the ClinPro Tools profiling purification kits from Bruker Daltonics.	
Magnetic particles with defined surface functionalities (magnetic  beadimmobilized metal	
ion affinity chromatography (MB-IMAC Cu), magnetic bead-hydrophobic interaction	
(MB-HIC C8) and weak cation exchange (MB-WCX)) were processed by the ClinPro	
Tools liquid handling robot according to the manufacturers protocol (Bruker Daltonics). 
Serum specimens were thawed on ice for 30 min and immediately processed according to	
our standardized protocol for serum peptidomics [55].	
Mass Spectrometry	
A linear MALDI-TOF mass spectrometer (Autoflex I, Bruker Daltonics) was used for	
the peptidome profiling. Daily mass calibration was performed using the standard	
calibration mixture of peptides and proteins in a mass range of 1-10 kDa. Mass spectra	
were recorded and processed using AutoXecute tool of the flexControl acquisition	
software (Ver. 2.0; Bruker Daltonics). The settings were applied as follows: Ion source	
1: 20 kV; ion source 2, 18.50 kV; lens, 9.00 kV; pulsed ion extraction, 120 ns;	
nitrogen-pressure, 2500 mbar. Ionization was achieved by a nitrogen laser (ë=337 nm)	
operating at 50 Hz.  For matrix suppression a high gating factor with    signal suppression	
up to 500 Da was used. Mass spectra were detected in linear positive mode.	
Baseline Removal	
The baseline is an exponential like offset dependent on the m/z value (mass-to-charge;	
x-value).  A baseline correction is performed to remove this rather   low-frequency noise	
from the spectrum. We use a morphological TopHat filter to eliminate  certain spatial	
structures within the signal, in our case the baseline. Note that this technique does not	
produce negative intensity values.	
